# Deep Learning Model-Based Architectures for Lung Tumor Mutation Profiling: A Systematic Review

**DOI:** 10.3390/cancers17223619

**Published:** 2025-11-10

**Authors:** Samanta Ortuño-Miquel, Reyes Roca, Cristina Alenda, Francisco Aranda, Natividad Martínez-Banaclocha, Sandra Amador, David Gil

**Affiliations:** 1 Alicante Institute for Health and Biomedical Research (ISABIAL), 03010 Alicante, Spain; 2 Department of Computer Science Technology and Computation, University of Alicante, 03690 Alicante, Spain; 3 Department of Pathology, Dr. Balmis General University Hospital, Alicante Institute for Health and Biomedical Research (ISABIAL), 03010 Alicante, Spain; 4 Department of Oncology, Dr. Balmis General University Hospital, Alicante Institute for Health and Biomedical Research (ISABIAL), 03010 Alicante, Spain

**Keywords:** deep learning, lung tumor, image classification, molecular profiling, explainability

## Abstract

**Background/Objectives**: Lung cancer (NSCLC), which accounts for approximately 85% of lung cancers, exhibits marked heterogeneity that complicates molecular characterization and treatment selection. Recent advances in deep learning (DL) have enabled the extraction of genomic-related morphological features directly from routine Hematoxylin and Eosin (H&E) histopathology, offering a potential complement to Next-Generation Sequencing (NGS) for precision oncology. This review aimed to evaluate how DL models have been applied to predict molecular alterations in NSCLC using H&E-stained slides. **Methods**: A systematic search following PRISMA 2020 guidelines was conducted across PubMed, Scopus, and Web of Science to identify studies published up to March 2025 that used DL models for mutation prediction in NSCLC. Eligible studies were screened, and data on model architectures, datasets, and performance metrics were extracted. **Results**: Sixteen studies met inclusion criteria. Most employed convolutional neural networks trained on publicly available datasets such as The Cancer Genome Atlas (TCGA) to infer key mutations including EGFR, KRAS, and TP53. Reported areas under the curve ranged from 0.65 to 0.95, demonstrating variable but promising predictive capability. **Conclusions**: DL-based histopathology shows strong potential for linking tissue morphology to molecular signatures in NSCLC. However, methodological heterogeneity, small sample sizes, and limited external validation constrain reproducibility and generalizability. Standardized protocols, larger multicenter cohorts, and transparent validation are needed before these models can be translated into clinical practice.

## Simple Summary

Lung cancer is a leading cause of cancer-related deaths worldwide, and understanding the genetic mutations that drive tumor growth is crucial for improving diagnosis and treatment. This study systematically reviews recent research using deep learning approaches to analyze lung tumor mutations, particularly in non-small-cell lung cancer. We summarize the architectures, data sources, and performance outcomes of various models, highlighting their potential for accurate and automated mutation profiling. The review also discusses current challenges, such as limited data and model interpretability, and identifies promising directions for future research. Our findings aim to guide scientists and clinicians in adopting deep learning techniques for more precise and efficient lung cancer genomics.

## 1. Introduction

Lung cancer is currently the most common cancer in men and the second most common cancer in women, being the cancer with the highest mortality rate worldwide for both sexes [1]. The two main histological types are non-small-cell lung cancer (NSCLC), which makes up 85% of cases, and small-cell lung cancer (SCLC), which accounts for the remaining 15% [2], the latter being more aggressive and characterized by rapid growth and development of metastases with increased resistance to both chemotherapy and radiotherapy [3]. Despite therapeutic advances, NSCLC remains aggressive, with 5-year survival rates of 63.7% for localized disease, 35.9% for locoregional disease, and only 8.9% for advanced stages [1]. These figures underscore the urgent need for improved outcomes.

The most common subtype of NSCLC is adenocarcinoma (40%), followed by squamous cell carcinomas (25%) and large cell carcinoma (10%), among others [4]. Moreover, the application of immunohistochemistry has allowed for greater accuracy in histological subclassification [5]. In contrast, the subclassifications of SCLC are small cell carcinoma and combined small cell carcinoma (SCLC with components of the histologic subtypes of NSCLC) [6,7].

Lung cancer is associated with smoking, air pollution (which is increasingly prevalent in society), and previous chronic lung diseases. It is important to note that, in the case of all diseases, the risk factor increases with aging due to telomere shortening and disruptions in DNA damage repair [8].

Technological advances in detection, digitization of images, development of new drugs and standardized protocols for methodology have enabled rapid detection, resulting in early treatment and an enormous impact on the patient’s quality of life and survival. In addition, molecular profiling using disease-specific gene panels or Next-Generation Sequencing (NGS) analysis is recommended in current clinical practice guidelines to help predict the patient’s clinical response and prognosis in order to develop the best treatment plan. Within lung cancer, the greatest advances in the knowledge of the different genomics mutations susceptible to targeted therapy have been developed in the adenocarcinoma subtype. Key biomarkers associated with targeted therapies include EGFR, ROS1, ALK, MET, RET, NTRK and KRAS [9,10]. Ongoing research continues to identify additional biomarkers, progressively broadening the spectrum of actionable treatment targets.

However, these techniques are costly and face several implementation challenges, including limited tissue availability, low sequencing quality, technical failures, and suboptimal results when analyzing paraffin-embedded samples using medical imaging software [11].

The role of artificial intelligence (AI), particularly deep learning (DL), is becoming increasingly significant in overcoming challenges related to lung cancer diagnosis and treatment. AI exhibits significant potential for analyzing complex histopathological and radiological images, enabling faster and more accurate classification of lung cancer subtypes [12]. For example, studies have demonstrated the effectiveness of DL algorithms in detecting subtle patterns within Hematoxylin and Eosin (H&E) stained images, which can predict molecular characteristics without the need for costly molecular testing, such as NGS [13,14]. AI models are also capable of integrating data from various sources, offering insights that complement conventional techniques and reduce diagnostic variability among pathologists [15]. Despite these significant advances, the key performance indicator (KPI) scores of current AI models have not yet reached the threshold expected in clinical practice for high-confidence use.

For all these reasons, it is essential to continue refining these technologies, improving their accuracy, and minimizing the risk of false negatives. These advances should also aim to reduce diagnostic time, ensure broader accessibility across pathology departments, and promote greater standardization to provide consistent results as the number of newly diagnosed patients continues to rise. DL offers the potential to develop more robust and efficient systems that enhance classification and prediction capabilities, improve mutational profiling, and reduce the workload for pathologists. Such progress could enable personalized treatment strategies, accelerate diagnostics, and ultimately improve patient outcomes.

The objective of this review is to critically assess the role of AI, particularly DL, in medical image analysis, with a specific emphasis on predicting molecular profiles of NSCLC from histopathological images. The novelty of this work lies in evaluating both the current state and future potential of AI-driven approaches for integrating image-based and molecular data. By focusing on the prediction of molecular profiles from H&E-stained images, this review aims to provide deeper insights into the molecular and cellular landscape of NSCLC. To date, this area remains insufficiently explored, particularly regarding key methodological aspects such as cross-validation, normalization, and explainability techniques. Addressing these factors is essential for developing a comprehensive and reproducible workflow that bridges image-based diagnosis, molecular prediction, personalized treatment, and prognosis.

In addition to synthesizing the main methodological trends, this review proposes a conceptual framework that summarizes best practices and recurring limitations to guide future standardization efforts in this emerging field.

## 2. Materials and Methods

For this systematic review, the instructions that were followed were the Preferred Reporting Items for Systematic Reviews and Meta-Analyses (PRISMA) 2020 [16]. This review was registered in the International Prospective Register of Systematic Reviews (PROSPERO; registration number CRD420251143921). The full protocol is available at https://www.crd.york.ac.uk/PROSPERO/view/CRD420251143921, registered on 1 October 2025.

### 2.1. Eligibility Criteria

This review will include peer-reviewed original research articles that specifically address non-small-cell lung cancer (NSCLC) with an emphasis on hematoxylin and eosin (H&E) imaging, molecular profiling, and the application of artificial intelligence (AI) algorithms. These criteria are selected to highlight methodologies capable of providing novel insights into the molecular and cellular mechanisms of NSCLC. Studies will be considered regardless of the reported patient demographics, such as age, gender, or ethnicity, as these factors are not the focus of the present review. Likewise, studies employing different image analysis techniques will be included, with image segmentation considered only when directly relevant to molecular profiling but not established as a mandatory prerequisite for eligibility. No restriction will be applied regarding the year of publication.

### 2.2. Exclusion Criteria

This review will be limited to articles written in English or Spanish, as the research team does not possess sufficient expertise in other languages, which ensures both accuracy of interpretation and methodological consistency. Studies addressing other carcinomas, including small-cell lung cancer (SCLC), will be excluded in order to maintain a strict focus on NSCLC. Research articles without full-text availability through institutional access (Dr. Balmis General University Hospital, Alicante Institute for Health and Biomedical Research (ISABIAL), and the University of Alicante) will also be excluded from consideration. Furthermore, only peer-reviewed original research will be included, while reviews (in accordance with PRISMA 2020 guidelines), preprints, dissertations, and theses will be omitted to guarantee methodological rigor and reliability of the findings. Finally, documents such as meeting abstracts, proceedings, or conference papers will be excluded, as they often represent preliminary or incomplete studies and may not provide the comprehensive data required for systematic evaluation.

### 2.3. Bias Assesment

This review shows several biases that may limit how well these models work across different populations. Differences in demographics, such as ethnicity, age and comorbidities, as well as variations in image processing techniques, like color normalization and patch selection, make results less consistent. Many studies do not always report important metrics, which help understand how models might perform in real-world settings. Variability in expert opinions, differences in sequencing methods, and inconsistent use of pathological staining add further challenges. Such variability can result in increased false positive and false negative rates, highlighting the need for standardized methodologies and detailed reporting to ensure generalizability across patient populations.

### 2.4. Information Sources

The reference databases Scopus, PubMed, and Web of Science (WOS) were electronically searched for eligible studies. The search was conducted in March 2025.

### 2.5. Search Strategy

Keywords for the search were selected according to the framework of the review. As primary concepts for the search, “lung tumor”, “deep learning”, “molecular profiling” and “hematoxylin and eosin” were selected. The resulting queries are shown below and are finally run on Scopus, PubMed and WOS, as they are the main world databases of bibliographic references.

**Scopus:** TITLE-ABS-KEY(”Lung cancer”) AND (TITLE-ABS-KEY(”deep learning”) OR TITLE-ABS-KEY(”Machine learning”) OR TITLE-ABS-KEY(”Artificial Intelligence”)) AND (TITLE-ABS-KEY(variant) OR TITLE-ABS-KEY(mutation*) OR TITLE-ABS-KEY(molecular) OR TITLE-ABS-KEY(molecular AND profil*)) AND TITLE-ABS-KEY(”Hematoxylin” AND ”eosin”)

**PubMed:** ((Lung cancer) AND (Deep learning OR Machine learning OR Artificial Intelligence) AND (variant or mutation* or molecular or (molecular AND profil*)) AND (Hematoxylin AND eosin))

**WOS:** (AB=(Lung cancer) AND AB=(Deep learning OR Machine learning OR Artificial Intelligence) AND AB=(variant or mutation* OR molecular OR (molecular AND profil*)) AND AB=(Hematoxylin AND eosin)) OR (TI=(Lung cancer) AND TI=(Deep learning OR Machine learning OR Artificial Intelligence) AND TI=(variant or mutation* OR molecular OR (molecular AND profil*)) AND TI=(Hematoxylin AND eosin))

### 2.6. Selection Process

After obtaining the results from the three databases, the tables were merged using the DOI URL as a common key to consolidate each record into a single entry. This process was conducted using R software (version 4.4.1) with the dplyr package (version 1.1.4). Subsequently, records were filtered by document type to exclude non-peer-reviewed materials—namely, meeting abstracts, patents, preprints, proceedings papers, conference papers, dissertations, and theses. Review articles were also excluded to minimize redundancy, in alignment with best practices for systematic reviews (script could be found on GitHub (https://github.com/Samantao93/NCSLC_systematic_review) accessed date 1 October 2025.

**Figure 1:**
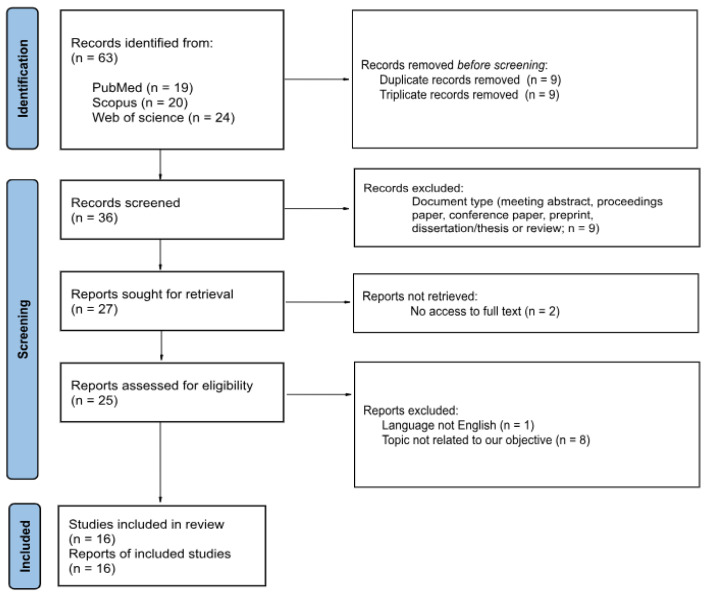
PRISMA 2020 Flow Diagram for Study Selection [16]. This figure summarizes the systematic selection process based on PRISMA 2020 guidelines. From an initial set of 63 articles, 16 were retained after excluding duplicates, non-peer-reviewed sources, non-English texts, and studies not focused on NSCLC molecular profiling via deep learning detailed in Section 2.3.

Next, in a manual search of the records, two of them were not open access, the language was different from English (Chinese), or those focusing on carcinomas different than NSCLC were excluded, retrieving a total of 16 articles. More detailed information is provided in Supplementary Information Table S1). This selection is summarized in Figure 1.

### 2.7. Data Items

This review examined critical aspects of DL model implementation, including model type (supervised or hybrid), predominant architectures, and the contribution of expert pathologists to segmentation and molecular profiling. We analyzed data sources (public, internal, or combined), dataset origins, and sample sizes, as well as the application of image normalization techniques. Validation strategies such as cross-validation and external testing were evaluated alongside data augmentation and explainability methods used for classification or mutational prediction. In addition, we assessed code and data availability to determine reproducibility and reported model performance metrics, including the Area Under the Receiver Operating Characteristic Curve (AUC), confidence intervals when available, and the biomarkers analyzed. All these variables are highly relevant, as they directly influence model robustness, generalizability, and predictive accuracy.

### 2.8. Data Collection Process

An Excel spreadsheet was created with different columns accordingly with the Section 3. All relevant information is extracted to acquire a global understanding on this topic (Supplementary information Table S1).

## 3. Results

### 3.1. Size of Population Used

**Figure 2:**
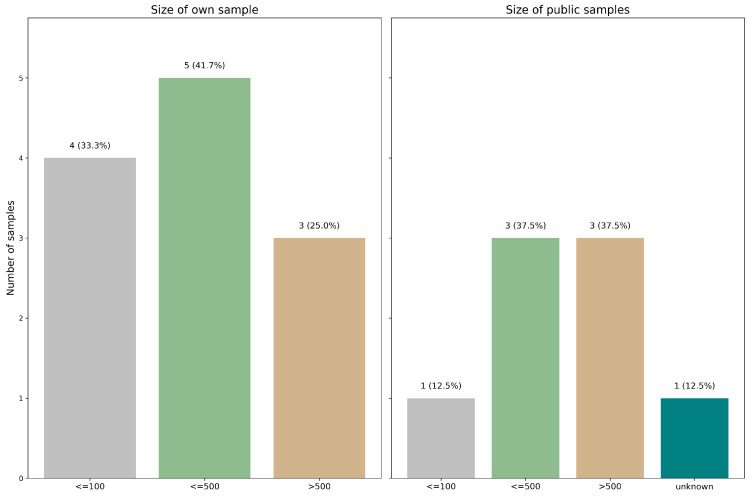
Distribution of dataset sizes across the reviewed studies. Studies were grouped by the number of samples used to train their deep learning models.

It is well-established that increasing the number of training slides generally leads to higher model accuracy. With this in mind, the total number of samples was divided into four groups, organized from lowest to highest statistical power as follows:

(1) Less than or equal to one hundred, constituting 33.3% (4/12) of the internal database [17,18,19,20] and 12.5% (1/8) of the public database [21]. (2) Less than or equal to five hundred but more than one hundred, comprising 41.7% (5/12) of the internal database [11,21,22,23,24], and 37.5% (3/8) of the public database [17,25,26]. (3) More than five hundred, making up 25.0% (3/12) of the internal database [22,25,27] and 37.5% (3/8) of the public database [28,29,30]. (4) Not known, accounting for 12.5% (1/8) of the public database [20] (see Figure 2).

The analysis was conducted using either internal or public databases to ensure a focused approach. Notably, a combination of databases was employed in some cases, resulting in superior outcomes compared to using a single data source.

### 3.2. Origin of the Own Samples

**Figure 3:**
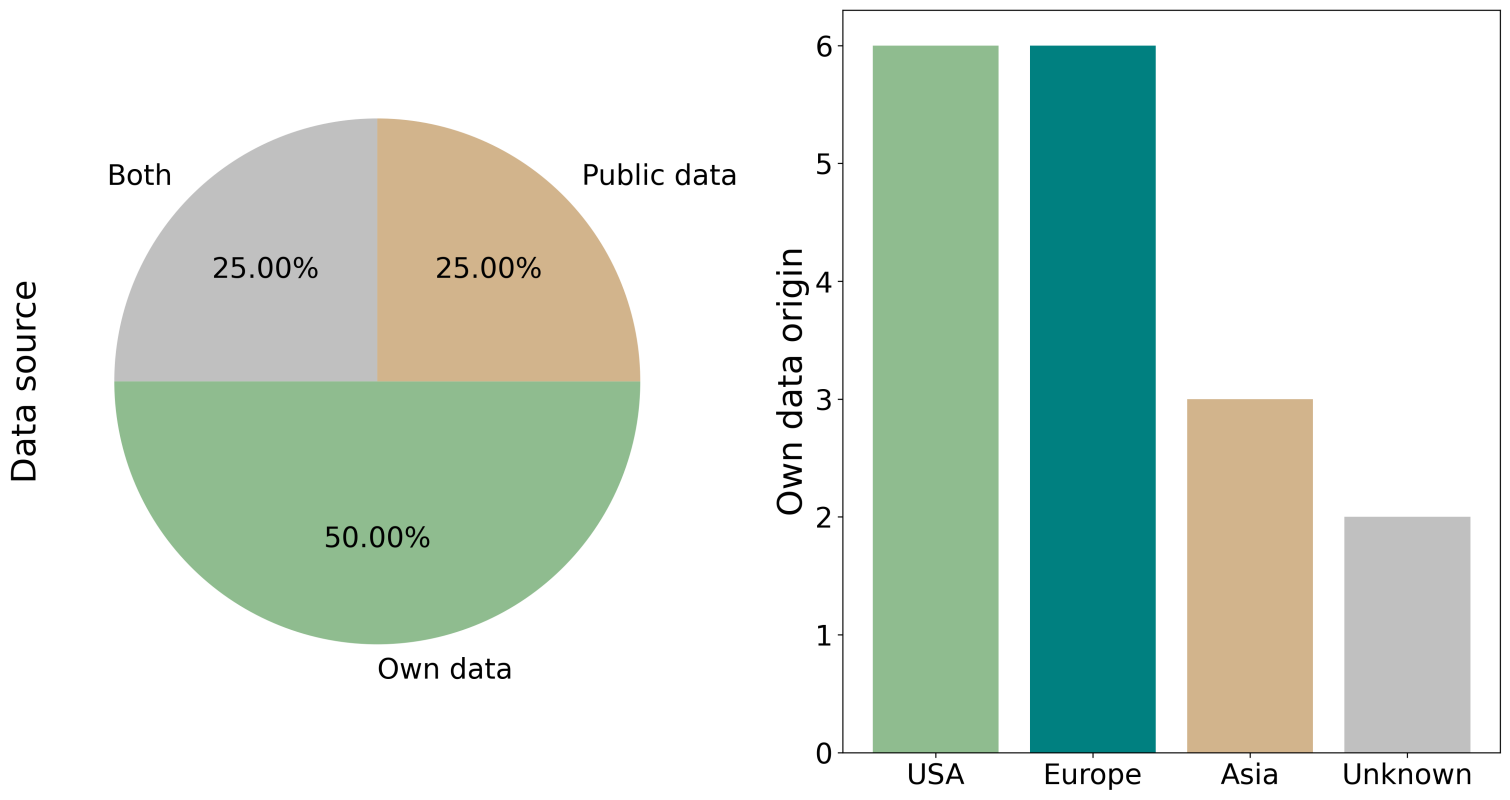
Source and geographic origin of the datasets used. Datasets were categorized as Public (e.g., TCGA, CPTAC), Own (institution-generated), or Mixed. Studies using both public and internal sources demonstrated improved model performance due to higher data heterogeneity.

The origin of the datasets was classified into three categories: Public data (including TCGA, CPTAC3, TRACERx, LATTICe-A and DHMC), Own data and Both. The most utilized dataset was Own data, accounting for 50.00% (8/16) of the studies [11,18,19,22,23,24,27,31]. The next categories was Both, comprising 25.0% (4/16) of the studies [17,20,21,25] and Public data at the same percentage (4/16) [26,28,29,30] (see Figure 3).

The reports with Own data (including those utilizing Both) originate from diverse regions. The distribution is as follows: the USA [18,20,21,22,25,31] and Europe (in article [22], two different databases from Europe are used) [17,21,22,24,31] each account for 37.5% (6/16) of the reports, followed by Asia at 18.7% (3/16) [11,18,23]. Additionally, 12.5% (2/16) of the reports are of unknown origin [19,27]. It is important to note that the total percentage exceeds 100% due to the utilization of multiple sources across different reports (see Figure 3).

### 3.3. Color and Other Normalization

**Figure 4:**
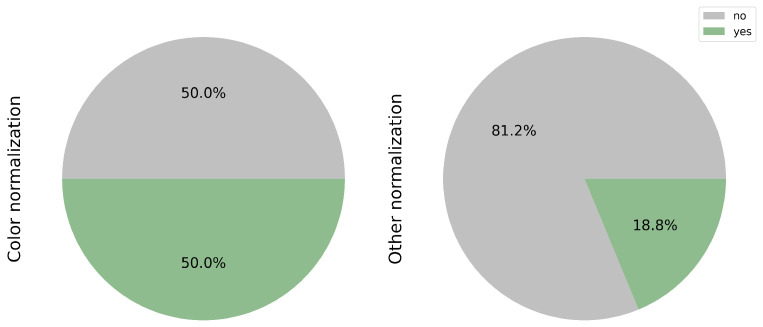
Implementation of color and other image normalization techniques. 50% of the reviewed studies applied color normalization to reduce scanner and staining variability. Additional preprocessing steps—such as magnification adjustment and background artifact removal—were employed in 18.8% of cases to improve feature extraction.

The significance of color normalization and other forms of normalization in achieving favorable outcomes is highlighted. Variations in protocols, tools, or technicians can impact the model’s interpretation, affecting its ability to detect pertinent features. In this context, color normalization was implemented in 50.0% (8/16) of the studies [17,18,22,24,26,27,30,31]. Additionally, other forms of normalization, such as adjusting the image magnification for analysis purposes or eliminating artifacts and background noise, were applied in 3 studies of 16, accounting for 18.8% [25,27,30] (see Figure 4).

### 3.4. Data Augmentation

**Figure 5:**
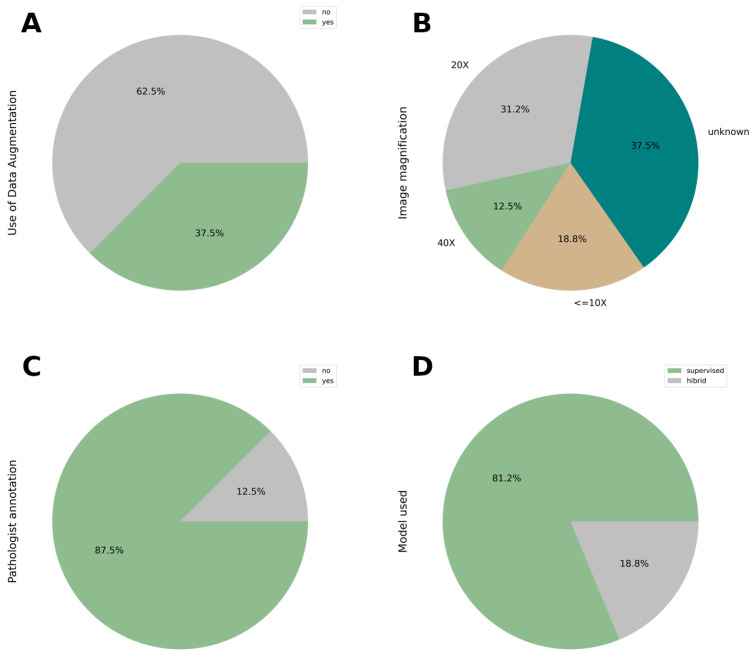
(**A**) Use of data augmentation techniques. Data augmentation was used in 35.7% of studies to increase training data diversity and mitigate overfitting. (**B**) Magnification Settings Used in Histological Image Analysis. Magnification varied across studies, ranging from 5X to 40X. 35.7% of articles did not specify the magnification used, raising concerns about reproducibility. Magnification can influence feature visibility and model performance. (**C**) The involvement of the expert pathologist in the annotation of the image. (**D**) Deep learning models applied in the context of this review.

The total number of slides used to train the model holds significant relevance, especially when the pool of patients available for analysis is limited. In such scenarios, the utilization of data augmentation emerges as a valuable strategy. By extending the training time and incorporating additional features, data augmentation enhances the model’s accuracy in achieving its primary objectives. This approach becomes particularly indispensable in enhancing the reliability of the results. However, only 6 out of the 16 papers, accounting for 37.5% [11,17,23,28,30,31], have implemented this technique (see Figure 5A).

### 3.5. Image Magnification Used

There is no criterion to achieve a better result, but the ranges go from 5x to 40x, except 1x used in the mouse model organism [19]. In the Figure 5B shows 18.8% (3/16; [19,20,25]) of the magnifications are lower than 10x, 31.2% (5/16; [18,23,24,27,28]) are 20x and 12.5% (2/16; [11,30]) are 40x. The other large percentage, 35.7% (6/16; [17,21,22,26,29,31]) is not describe in the article.

### 3.6. Expertise Pathologist

At this moment of the development worldwide, the figure of the expert pathologist is still necessary for the annotation of the image. It is reflected in Figure 5C; 14 articles (representing 87.5%) need expert criteria to develop the model and categorize the classification of the slide, except 2, representing 12.5% [25,28].

### 3.7. Deep Learning Architectures: Supervised vs. Hybrid Approaches

Among the reviewed studies, supervised deep learning (DL) models were the predominant choice (81.2% (13/16) [17,18,19,21,22,23,24,26,27,28,29,30,31]) while a smaller portion (18.8% (3/16) [11,20,25]) employed hybrid approaches that combined supervised and unsupervised techniques. The selection of model architecture typically depends on the specific task—whether image classification or mutation profiling (Figure 5D).

Supervised models, such as convolutional neural networks (CNNs), ResNet variants, and Inception v3, require labeled input data (e.g., tumor vs. non-tumor annotations by pathologists). These architectures are well-suited for histological classification tasks, where morphological features are visually discernible. ResNet, in particular, is favored for its skip connections that alleviate the vanishing gradient problem and facilitate the training of deep networks.

In contrast, hybrid models integrate both supervised learning (e.g., classification of tissue types) and unsupervised methods (e.g., clustering of image features or dimensionality reduction using autoencoders). These models are especially useful in mutation profiling, where the visual patterns linked to specific genetic alterations may not be easily annotated. Hybrid approaches allow the model to discover latent patterns and relationships between image data and molecular profiles.

### 3.8. Architectures Used for Classification and Mutational Profiling

In current workflows for computational pathology in lung cancer, a typical pipeline begins with the classification of histological slides, frequently distinguishing healthy from tumor tissue. This task often depends on expert pathologist annotations and guides subsequent steps in automated analysis. Once tumor regions are localized, DL models are employed to characterize the mutational landscape of each patient. Accurate sequencing, preprocessing, and normalization of raw data are crucial to ensure robust mutation detection.

**Figure 6:**
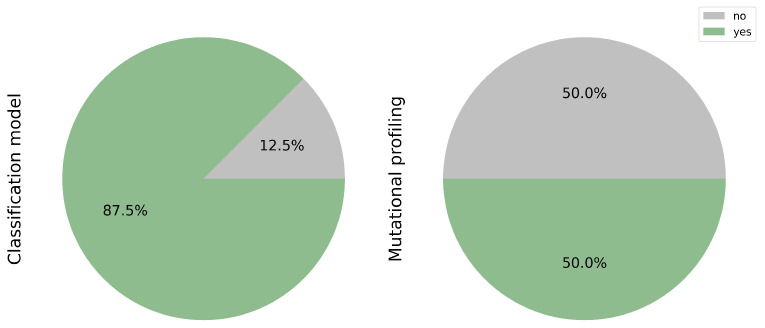
Tasks addressed: Classification and Mutation Profiling. While 85.7% of studies applied deep learning for histological classification, only half extended their analysis to mutation profiling. This gap highlights the opportunity to further integrate molecular prediction in computational pathology.

Across the studies included in the systematic review, a strong preference is observed for slide classification models. Specifically, 85.7% (14 out of 16 studies; [17,18,19,20,21,22,23,24,25,26,27,29,30,31]) employed deep learning for histopathological classification. Conversely, mutation profiling was implemented in 50.0% of the studies (8 out of 16; [11,17,23,24,25,26,27,28]), as illustrated in Figure 6.

Table 1 and Table 2 provide a summarized overview of the deep learning architectures used across both classification and mutational profiling applications.

ResNet-based models are widely adopted in both classification and mutation prediction tasks. Notably, the ANORAK architecture, which incorporates pyramid pooling and cross-stream attention mechanisms, was used for histopathological grading of lung adenocarcinoma [30]. Other studies apply ResNet variants for EGFR mutation prediction and histological classification [23,25,27].

CNNs remain fundamental in many frameworks, supporting both classification [17,26,29] and mutational profiling tasks [26]. Furthermore, advanced feature extraction techniques such as the Graph-based Sparse PCA Network (GS-PCA) have been proposed for segmentation and interpretability tasks [19].

Additional models include Inception v3 [20,31] and DeepLab v3 [21,23], which are used primarily for classification. Commercial platforms like Lunit SCOPE have also gained traction for automated H&E slide interpretation [29].

### 3.9. Gene Analyzed

These biomarkers are concluded to be essential to determine the personalized treatment that could result in a better progress and survival of the patient. In Table 2 could be seen that the gene that are more studied is *EGFR* [17,23,25,27] with different precision in the analysis (Area Under the Curve (AUC) from 0.65 to 0.87; none of the studies reported the 95% confidence interval). Other genes establish as recommended to this type of carcinoma are also analyzed. Mutations in genes as *BRAF*, *KRAS*, *MET*, translocations such as *ALK*, *ROS1*, *RET* or the protein expression of PD-L1. Another emerging biomarkers such as *STK11* and *KEAP1* are gaining attention, albeit not as extensively studied as others. Notably, certain biomarkers such as HER2 and NTRK have yet to receive significant scrutiny in deep learning studies for lung cancer. HER2 alterations, which may arise from amplification, overexpression, or activating mutation, have been reported but remain underexplored in DL-based studies due to their variable biological and clinical implications and low prevalence in NSCLC. Additionally, these alterations often require molecular assays like FISH or RNA-seq for accurate detection, limiting the feasibility of learning them directly from H&E slides. The lack of distinguishing histological features and clinical prioritization further reduce their representation in public datasets. Addressing this gap will require novel approaches such as multimodal learning, data augmentation or transfer learning to enable robust prediction despite data imbalance.

### 3.10. Cross-Validation or External Data Used for Model Validation

**Figure 7:**
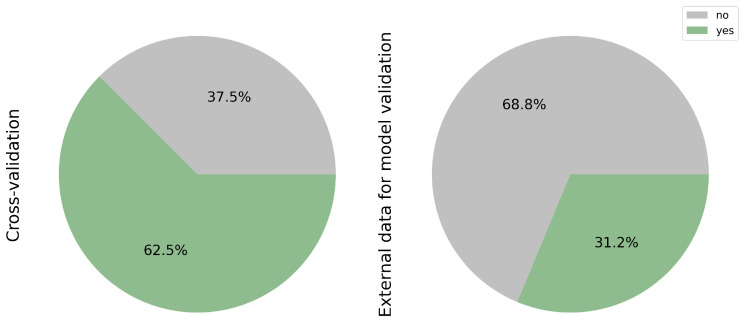
Validation methods: cross-validation vs. external datasets. Most studies relied on internal cross-validation (62.5%), with only 31.2% incorporating independent external cohorts. External validation is crucial to assess model robustness and real-world applicability.

While cross-validation remains a prevalent technique in AI, it is imperative to recognize its limitations. Training and validating a model on the same dataset may lead to reduced accuracy when applied to new datasets. Incorporating external data offers a more robust approach by introducing greater variability, thereby assessing the model’s adaptability to diverse patient populations. Figure 7 underscores the widespread utilization of cross-validation, accounting for 62.5% of surveyed studies (10/16) [11,17,18,19,20,24,26,27,28,30] compared to the external validation dataset representing 31.2% (5/16) [20,21,22,25,31]. This discrepancy highlights an opportunity for greater integration of external datasets in AI model development to enhance generalizability and robustness across patient cohorts.

### 3.11. Explainability of the Classification and Mutational Profiling Model

**Figure 8:**
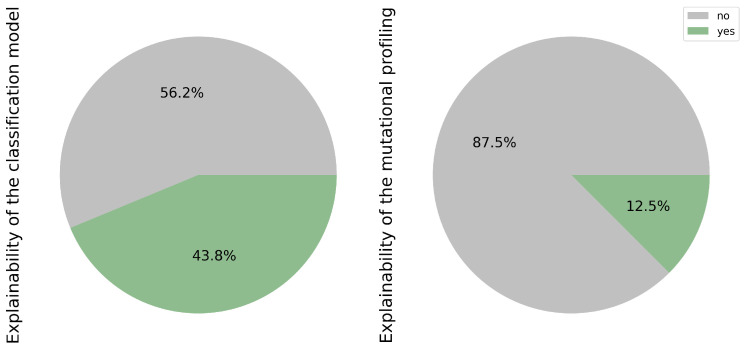
Explainability used for the outcome of the classification and mutational profiling model.

While machine learning models boast high accuracy, their lack of transparency and interpretability poses challenges in understanding the factors influencing their decisions. Consequently, there is a growing emphasis within the data science community on enhancing model explainability. Figure 8, shows the classification model on 43.8% of the articles (7/16) [17,19,20,22,23,24,26]. These efforts aim to elucidate the critical factors driving model decisions. In contrast, only 2 out of the total number of articles (12.5%) addressing mutational profiling addressed this issue [23,28], representing the grade of *EGFR* mutation rates and various mutational classes using a heatmap generated from Whole Slide Imaging (WSI).

This discrepancy underscores the relative lack of attention to explainability within the context of mutational profiling.

### 3.12. Open Data and Code

**Figure 9:**
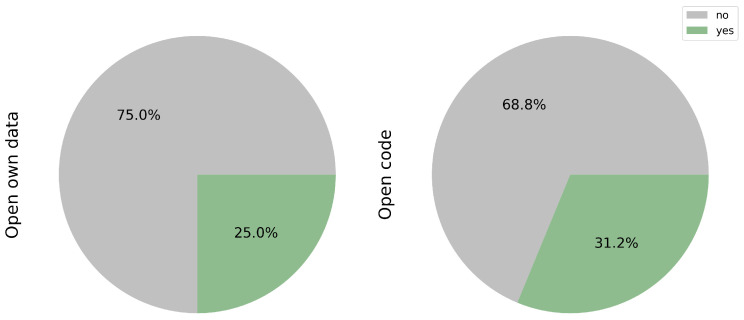
Publicly available open data and code.

Open science is increasingly important to achieve greater robustness in research and not to repeat certain studies that are widely conducted. For this reason, achieving full transparency, particularly concerning open data and researchers’ code, remains a challenge, as highlighted in Figure 9. This challenge may stem from difficulties in controlling the authority of publications and ensuring the commitment of research groups, both in terms of time and financial resources, to sharing their data and code.

While public databases offer valuable resources for researchers, only a fraction of the studies reviewed have shared their own data, accounting for 25.0% of the total (3/12) [17,18,23]. Access to such data could significantly enhance training datasets, thereby increasing sample variability. However, prior scrutiny of the protocols used, particularly in mutational profiling, is essential to mitigate potential biases in both the sample and prediction models.

Equally crucial is the sharing of code, as it facilitates model optimization and expedites the implementation of transfer learning techniques. Notably, only 31.2% (5/16) of the studies reviewed have made their code publicly available [17,18,23,28,30].

Efforts to encourage greater openness and transparency in research practices are pivotal for advancing scientific knowledge and fostering collaboration within the research community.

### 3.13. Other Relevant Information

All the studies analyzed have utilized WSI. This imaging technique captures extensive information about the tumor in a single slice, not only detailing the tumor itself but also its surrounding microenvironment. This holistic perspective enhances our understanding of pathology. The future approach will focus on WSI, as it represents raw clinical data obtained directly from routine procedures. The proposed model aims to streamline the process, reducing both time and additional preprocessing steps required to generate results.

Among the algorithms analyzed, all are based on human data except for one, which employs a mouse model as its organism [19]. Given the capabilities of DL algorithms, it is well recognized that using human imaging data enhances accuracy and aligns with precision medicine principles. The efforts will be directed toward achieving optimal results based on human-derived data and WSI.

Alternative approaches unrelated to our molecular profiling method include Tumor Mutational Burden (TMB) analysis, which quantifies the number of mutations per megabase of DNA and serves as a predictive biomarker for immunotherapy response [18]. Tumor Cellularity (TC) estimation assesses the proportion of tumor cells within a sample, which is crucial for evaluating tumor purity and ensuring the accuracy of genomic analyses [21]. Tumor-Infiltrating Lymphocyte (TIL) scoring evaluates immune cell infiltration within the tumor microenvironment, providing valuable prognostic insights and guiding treatment decisions [22]. Additionally, Immune Checkpoint Inhibitor (ICI) response assessment predicts a tumor’s likelihood of responding to immunotherapy by analyzing immune-related markers [31].

## 4. Discussion

Model selection in deep learning (DL) depends on task complexity, data availability, heterogeneity, and the intended clinical application. Supervised models achieve high accuracy with well-annotated data, while hybrid architectures exploit unlabeled or weakly labeled information—especially valuable for rare mutations and multimodal integration (e.g., histology and genomics). These hybrid and multimodal approaches are expected to drive the next generation of scalable and explainable DL systems in precision oncology.

DL algorithms have been widely applied to classify lung cancer using computed tomography (CT), positron emission tomography (PET), and magnetic resonance imaging (MRI) for tumor detection and localization [32,33,34]. Recently, hematoxylin and eosin (H&E)-stained whole slide images (WSI) have gained attention as accessible and information-rich data sources. Proper color normalization and robust architecture design enable H&E images to achieve high predictive accuracy for mutation profiling, prognosis, and treatment selection [37,38]. Integrating these images into DL pipelines therefore offers both practical and clinical advantages.

This review identified only sixteen studies addressing molecular profiling of non-small-cell lung cancer (NSCLC) from routine H&E slides, highlighting the early stage of research in this domain. Most datasets included roughly 500 samples—adequate for experimental models but insufficient for clinical translation. Public repositories help expand sample size, though their curation may reduce real-world variability. Nearly all studies analyzed human samples, emphasizing the need for accurate, clinically relevant models that can enhance patient outcomes and reduce costs by minimizing unnecessary procedures.

H&E WSIs were the most frequent data type, yet no standardized image augmentation or normalization protocols were observed. These processes are critical for harmonizing color, scale, and contrast, particularly when combining institutional and public data. Data augmentation, used in fewer than half of the studies, remains essential for addressing class imbalance and improving generalization, especially for rare variants. External validation—performed in only 31% of studies—should replace exclusive reliance on cross-validation to ensure true generalizability.

A major limitation across current deep learning (DL)–based studies is the limited control for confounding clinical and demographic variables such as ethnicity, smoking status, age, and sex, which are strongly correlated with oncogenic drivers in NSCLC. For instance, the prevalence of EGFR mutations is markedly higher in East Asian populations (38–49%) compared with Western cohorts (11–33%) [39]. Smoking status is another crucial determinant, as EGFR and ALK alterations are enriched in never-smokers, while KRAS—particularly KRAS-G12C—is strongly associated with tobacco exposure [40]. Likewise, ALK and ROS1 fusions are more frequent among younger, non-smoking females [41]. When models are trained without stratification or adjustment for these covariates, their apparent accuracy may reflect patient-mix effects or acquisition biases rather than genuine genotype-specific morphology. Recent evidence shows that DL models can even infer demographic traits such as race from histopathological or radiological images, underscoring the risk of shortcut learning [42]. Therefore, minimum safeguards should include patient-level and site-stratified splits with external validation, multivariable adjustment for demographic factors, explicit reporting of mutation subtypes (e.g., KRAS-G12C vs. non-G12C), and harmonization procedures to mitigate batch effects. Future work should combine multimodal data (histopathology + genomics + clinical) and implement explainable AI techniques to disentangle these effects and ensure biological interpretability [43].

Although molecular profiling through NGS or targeted sequencing represents the current gold standard, these analyses remain relatively expensive, time-consuming, and resource-intensive. They require sufficient tumor tissue, specialized technicians, and expert molecular geneticists to interpret variants, coverage, and fusions, which increases the turnaround time and overall cost.

In contrast, DL-based approaches could provide a cost-effective and rapid complementary strategy, especially in cases with scarce tissue or limited access to molecular testing facilities. A reliable DL model capable of inferring the likelihood of actionable mutations directly from H&E slides could help prioritize samples for confirmatory testing, reduce diagnostic workload, and optimize healthcare resources. However, these systems should not replace molecular diagnostics, and any AI-based prediction must be confirmed by validated assays before therapeutic decisions are made.

Expert pathologists were consistently involved in segmentation and classification, confirming that human oversight remains indispensable, even when models output mutation predictions. Supervised approaches dominate classification tasks, whereas mutational profiling increasingly integrates semi- or unsupervised components.

Transfer learning appeared as a common strategy to boost accuracy while reducing computation. Architectures such as ResNet, U-Net, and CNN variants are widely used, while transformer-based and multimodal fusion networks are emerging for precision oncology. XAI techniques—Grad-CAM, LIME, and SHAP—support model transparency [44], yet only half the reviewed works applied them for classification and fewer than 15% for mutation prediction, revealing a key gap between performance and interpretability.

Reported Area Under the Curve (AUC) values varied by gene and architecture: EGFR (0.65–0.87), TP53 (0.71–0.80), and fusions such as ALK or ROS1 reaching near-perfect accuracy (AUC = 0.99–1.0) [11,23,25]. This variability underscores the influence of model design, dataset size, and preprocessing on performance and highlights the absence of standardized benchmarks.

**Figure 10:**
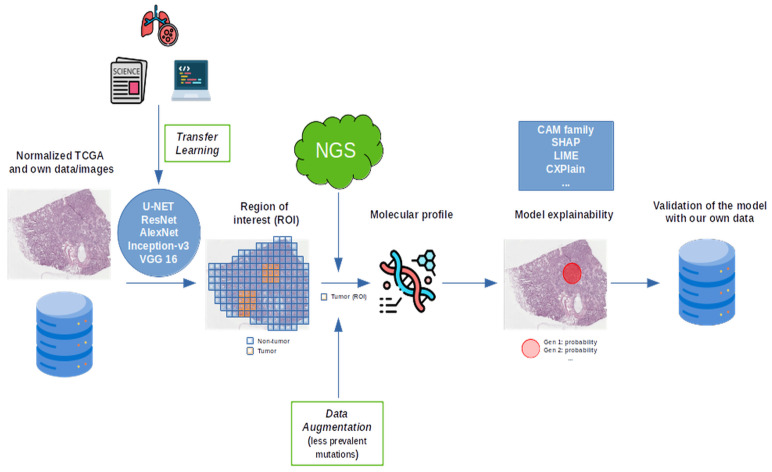
A schematic representation of the future approach.

In summary, DL models for molecular prediction in lung cancer show strong potential but remain limited by small datasets, inconsistent validation, and poor explainability. Future progress depends on larger, diverse cohorts, transparent algorithms, and external evaluation. Based on the synthesis of methodologies identified across the reviewed studies, we propose a conceptual multimodal AI framework (Figure 10) to illustrate how a more robust and standardized pipeline could be achieved. This proposal does not represent a newly developed algorithm but rather integrates the most recurrent components and best practices observed in previous works—such as hybrid supervision, transfer learning, and explainability modules—into a unified workflow. The framework is intended to serve as a guide for future studies aiming to enhance reproducibility, interpretability, and clinical applicability of DL models in NSCLC molecular profiling.

## 5. Conclusions

This paper presents a comprehensive review of DL models in NSCLC mutation profiling using H&E images. Although this field remains in its early stages, this analysis highlights the promising integration of H&E imaging with molecular profiling to advance the understanding of lung cancer. We also identify methodological discrepancies—including limited dataset diversity, lack of external validation, and insufficient explainability—which represent opportunities to refine model architectures and promote more standardized, reproducible pipelines in this area.

Looking ahead, our research aims to integrate multimodal AI-based solutions into clinical workflows for the predictive analysis of molecular alterations using digitized H&E-stained tumor images. The objective is to improve diagnostic accuracy and establish these models as efficient pre-screening tools prior to more resource-intensive molecular assays. By combining histopathological imaging, genomic profiles, and clinical metadata within a unified multimodal learning framework, we seek to capture complementary biological and contextual information that enhances predictive precision, early detection, and patient stratification for targeted therapies. This integrated approach—grounded in multimodal data fusion and explainable deep learning—promises to streamline clinical decision-making, support precision oncology, and ultimately improve patient outcomes in lung cancer diagnosis and treatment.

## Figures and Tables

**Table 1:** Deep learning models used for histopathological classification in lung cancer.

Reference	Model Architecture	Classification Task
[2]	Probabilistic fusion (RNA-Seq + histology)	NSCLC subtype classification using multimodal data
[12]	ResNet50 (custom)	Slide-level classification of lung cancer
[21]	DeepLab v3+	Tumor cellularity classification from IHC-restained slides
[32]	KPCA + CNN + DBNN	Classification of lung cancer from image features
[33]	Multiple CNN architectures (review)	Overview of detection/classification methods for lung cancer
[34]	ResNet, VGG, AlexNet (review)	Survey of deep learning techniques in lung cancer diagnosis
[35]	CNN (unspecified)	Prediction of cancer biomarkers from tissue slides
[36]	CNN (custom)	Prognosis and treatment response prediction in SCLC

**Table 2:** Deep learning models and their performance for mutational profiling in lung cancer.

Reference	Model Architecture	Mutational Task	AUC
[11]	CNN (unspecified)	Direct identification of *ALK* and *ROS1* fusions from H&E	1.000, 0.986
[17]	CNN + attention	Segmentation of tumor microenvironment predictive of mutation and survival (*RET* *, *KRAS* *, *KEAP1* *, *TP53* *, *BRAF*, *PDGFRB*, *ROS1* *, *STK11* *, *MET*, *ALK* *, *DDR2*, *PIK3CA* *, and *EGFR* *)	0.67, 0.62, 0.61, 0.59, 0.57, 0.74, 0.61, 0.63, 0.69, 0.63, 0.57, 0.58, 0.65
[23]	DeepLab v3	Prediction of *EGFR* mutation from H&E whole slide images	0.833
[25]	ResNet	Prediction of *EGFR* and *TP53* mutations from WSI	0.799, 0.713
[26]	Custom CNN	Prediction of drug-targetable gene mutations (*AKT*, *FGFR1*, *FGFR2*, *MET*) and *HRAS*	0.72, 0.83, 0.82, 0.86 and 0.79
[27]	ResNet18	Prediction of *EGFR* mutational status from pathology images	0.870

* The best results were obtained with models using a random forest classifier. The remaining models, in the same study, applied logistic regression.

## Data Availability

The original contributions presented in this study are included in the article/Supplementary Material. Further inquiries can be directed to the corresponding author. The code used in this study is openly available at GitHub (https://github.com/Samantao93/NCSLC_systematic_review).

## Supplementary Materials

The following supporting information can be downloaded at: https://www.mdpi.com/article/10.3390/cancers17223619/s1, Table S1: This table provides a step-by-step summary of the article selection process to enhance reproducibility and transparency.

## Abbreviations

The following abbreviations are used in this manuscript:

AI	Artificial Intelligence
AUC	Area Under the roc Curve
CAM	Class Activation Mapping
CNNs	Convolutional Neural Networks
DL	Deep Learning
FISH	Fluorescence In Situ Hybridization
Grad-CAM	GRADient-weighted Class Activation Mapping
GS-PCA	Graph-Based Sparse Principal Component Analysis
H&E	Hematoxylin and Eosin
ICI	Immune Checkpoint Inhibitor
IHC	ImmunoHistoChemistry
Indels	INsertions or DELetions
KPI	Key Performance Indicator
LIME	Local Interpretable Model-agnostic Explanations
MNVs	Multiple Nucleotide Variants
NGS	Next-Generation Sequencing
NSCLC	Non-Small-Cell Lung Cancer
PRISMA	Preferred Reporting Items for Systematic Reviews and Meta-Analyses
SCLC	Small-Cell Lung Cancer
SHAP	SHapley Additive exPlanations
SNVs	Single Nucleotide Variants
TC	Tumor Cellularity
TIL	Tumor-Infiltrating Lymphocyte
TMB	Tumor Mutational Burden
WOS	Web of Science
WSI	Whole Slide Imaging
XAI	eXplainable Artificial Intelligence
